# The geographic distribution of onchocerciasis in the 20 participating countries of the African Programme for Onchocerciasis Control: (2) pre-control endemicity levels and estimated number infected

**DOI:** 10.1186/1756-3305-7-326

**Published:** 2014-07-22

**Authors:** Honorat GM Zouré, Mounkaila Noma, Afework H Tekle, Uche V Amazigo, Peter J Diggle, Emanuele Giorgi, Jan HF Remme

**Affiliations:** 1 African Programme for Onchocerciasis Control, Ouagadougou BP 549, Burkina Faso; 2 Consultant, Box 3397, Main Post office, Enugu, Nigeria; 3 Medical School, Lancaster University, Lancaster, UK; 4 Institute of Infection and Global Health, University of Liverpool, Liverpool, UK; 5 Faculty of Health and Medicine, Lancaster University, Lancaster, UK; 6 Consultant, 120 Rue des Campanules, Ornex 01210, France

**Keywords:** Onchocerciasis, APOC, Onchocercal nodule, Mapping, REMO, Geostatistics, Endemicity level

## Abstract

**Background:**

The original aim of the African Programme for Onchocerciasis Control (APOC) was to control onchocerciasis as a public health problem in 20 African countries. In order to identify all high risk areas where ivermectin treatment was needed to achieve control, APOC used Rapid Epidemiological Mapping of Onchocerciasis (REMO). REMO involved spatial sampling of villages to be surveyed, and examination of 30 to 50 adults per village for palpable onchocercal nodules. REMO has now been virtually completed and we report the results in two articles. A companion article reports the delineation of high risk areas based on expert analysis. The present article reports the results of a geostatistical analysis of the REMO data to map endemicity levels and estimate the number infected.

**Methods:**

A model-based geostatistical analysis of the REMO data was undertaken to generate high-resolution maps of the predicted prevalence of nodules and of the probability that the true nodule prevalence exceeds the high risk threshold of 20%. The number infected was estimated by converting nodule prevalence to microfilaria prevalence, and multiplying the predicted prevalence for each location with local data on population density. The geostatistical analysis included the nodule palpation data for 14,473 surveyed villages.

**Results:**

The generated map of onchocerciasis endemicity levels, as reflected in the prevalence of nodules, is a significant advance with many new endemic areas identified. The prevalence of nodules was > 20% over an area of 2.5 million km^2^ with an estimated population of 62 million people. The results were consistent with the delineation of high risk areas of the expert analysis except for borderline areas where the prevalence fluctuated around 20%. It is estimated that 36 million people would have been infected in the APOC countries by 2011 if there had been no ivermectin treatment.

**Conclusions:**

The map of onchocerciasis endemicity levels has proven very valuable for onchocerciasis control in the APOC countries. Following the recent shift to onchocerciasis elimination, the map continues to play an important role in planning treatment, evaluating impact and predicting treatment end dates in relation to local endemicity levels.

## Background

Onchocerciasis, or river blindness, used to be endemic in some 30 countries in Africa where over 99% of all cases in the world were found [1]. The Onchocerciasis Control Programme in West Africa (OCP) has successfully controlled onchocerciasis by large scale vector control in the savanna belt of nine West African countries [2]. In the remaining endemic African countries, where some 85% of onchocerciasis cases lived, onchocerciasis control became feasible with the registration of ivermectin for the treatment of human onchocerciasis in 1987 and its donation free of charge for as long as needed [3,4]. Clinical and community trials demonstrated that annual ivermectin treatment could effectively control the disease [5], and Non-Governmental Development Organizations initiated the first ivermectin distribution efforts [6]. In 1995 the African Programme for Onchocerciasis Control (APOC) was created to support the establishment of community directed treatment with ivermectin (CDTI) in all remaining areas in Africa where onchocerciasis was a public health problem [7].

One of the first challenges for APOC was to determine where exactly onchocerciasis was a public health problem. The existing information on the geographic distribution of onchocerciasis in the 20 APOC countries [1,8-32] was incomplete and not reliable enough for targeting ivermectin treatment programmes, and there was an urgent need for comprehensive mapping of the geographic distribution of onchocerciasis in all potentially endemic countries in Africa outside the OCP [7,33]. This was a vast area of some 14 million km^2^ and the survey methods available were difficult to implement at such a large scale. In anticipation of this problem, the WHO Special Programme for Research and Training in Tropical Diseases (TDR) developed a rapid assessment method in 1993, Rapid Epidemiological Mapping of Onchocerciasis (REMO) [34]. In REMO, sample villages are selected using a sampling methodology that takes the spatial epidemiology of onchocerciasis into account. Rapid assessment surveys are then carried out in the selected villages to estimate the prevalence of palpable onchocercal nodules as a proxy for the prevalence of onchocerciasis infection. Following its successful field testing in Cameroon and Nigeria [35], APOC adopted REMO for large-scale mapping of onchocerciasis in all APOC countries in order to identify priority areas for CDTI. Large scale application of REMO started in 1996, and has since been applied in phase with the expansion of CDTI to cover all potentially endemic areas in APOC countries [33].

To date, virtually all potentially endemic areas in the 20 APOC countries have been mapped for onchocerciasis through REMO. In a companion paper we summarize the REMO surveys and show the results of an expert analysis that was undertaken to delineate high-risk areas where onchocerciasis was a major public health problem and where ivermectin treatment was a priority [36]. Based on these maps of high risk areas, CDTI projects were created that by 2012 were treating over 80 million people in the APOC countries [37].

In the present article we report the results of a more advanced analysis of the REMO data using a model-based geostatistical methodology that has allowed a more effective utilization of the extensive REMO data. One important application was the mapping of the geographic distribution of onchocerciasis endemicity levels as reflected in the prevalence of palpable onchocercal nodules. Endemicity is a key concept in onchocerciasis epidemiology. The severity of the disease and the public health importance of onchocerciasis in a given area are directly related to the local level of endemicity [38,39]. The endemicity level is also an important indicator of the local potential for transmission, as well as a predictor of the intensity and duration of interventions needed to control or eliminate onchocerciasis in an onchocerciasis focus [40]. It is therefore important for onchocerciasis control programmes to have a detailed map of onchocerciasis endemicity levels throughout their operational area.

In 1979, Prost *et al*. [41] defined three levels of onchocerciasis endemicity in terms of the community prevalence of *Onchocerca volvulus* microfilaria in the skin: hyperendemic onchocerciasis (prevalence of microfilaria > 60%),where the disease is very severe and associated with onchocercal blindness rates in excess of 4 to 5% in the West African savanna; hypoendemic onchocerciasis (prevalence of microfilaria <35%) where ocular complications are rare and the disease is socially not apparent, and mesoendemic onchocerciasis (prevalence of microfilaria between 35% and 60%) where the disease pattern varied between these two extremes. The prevalence of nodules is related to the prevalence of skin microfilaria. Using the quantification of this relationship by Coffeng *et al.*[42], the above endemicity classes translate into hyperendemic onchocerciasis for a prevalence of palpable nodules in adults > 45%, mesoendemic onchocerciasis for a nodule prevalence between 20% and 45%, and hypoendemicity for a prevalence of nodules < 20%.

When ivermectin became available for onchocerciasis control, a WHO expert meeting recommended that in order to control onchocerciasis as a public health problem, ivermectin treatment was urgent in communities with a prevalence of nodules in adult males > 40% and highly desirable for a nodule prevalence > 20%, i.e. in all meso and hyper endemic communities [43]. Based on this recommendation, APOC’s aim was to establish CDTI in all high risk areas where the prevalence of palpable nodules in adults was greater than 20% [33]. A first application of the geostatistical analysis was to delineate all areas where the estimated prevalence was > 20% and to compare the results with the classification of high risk areas from the expert analysis as reported in the companion paper. We also used the geostatistical analysis to provide population estimates by endemicity level, and to predict how many people would have been infected with *O. volvulus* in the APOC countries if there had been no onchocerciasis control.

## Methods

### REMO methodology

The geographic distribution of onchocerciasis is determined by the availability of breeding sites for the *Simulium* vectors in fast flowing rivers and streams, and the limited flight range of the vector when seeking a blood meal. The spatial epidemiology of onchocerciasis is therefore closely related to the distribution and suitability of local river systems. REMO is based on this knowledge and consists of three stages [34]:

1) The division of the area to be mapped into biogeographic zones that are reasonably uniform with regard to their potential for onchocerciasis and that cover the watersheds of the main local drainage systems. Areas that are known to be unsuitable for the vector for ecological reasons (absence of fast flowing water, high altitude, etc.) and uninhabited areas (e.g. national parks) are excluded at this stage.

2) The selection of a sample of villages to be surveyed in order to determine whether onchocerciasis is present or not and, if present, to give a rough indication of the distribution and severity of onchocerciasis in the zone. This sampling uses the available information on the local river system

3) Rapid epidemiological assessment (REA) surveys in the selected villages. A sample of 30 to 50 adult males who have resided in the village for more than 10 years are examined for the presence of nodules, and the percentage of males with palpable nodules is calculated. The geographic coordinates of each village are collected by applying a Global Positioning System (GPS) in a central location in the village.

More details of the REMO methodology are provided in the companion paper [36] and the WHO Manual for Rapid Epidemiological Mapping of Onchocerciasis [34]. The companion paper also describes the implementation of REMO in APOC countries and ethical considerations in undertaking the REMO surveys.

### Analysis of REMO data

The analysis of the REMO data was undertaken using two analytical approaches: an expert analysis using the original REMO analytical methodology for which the results are reported in the companion article [36], and a geostatistical analysis which is described here.

### Geographic information system (GIS)

All relevant geographic information was processed using ArcGIS 10 (ESRI Inc., Redlands, USA).

The geographic information used for the analysis included:

– National and administrative boundaries, rivers and lakes, national parks, main roads, villages and urban settlements (source WHO HealthMapper http://health-mapper-release-5.software.informer.com).

– Topography and relief (source ESRI http://services.arcgisonline.com/ArcGIS/rest/services/World_Shaded_Relief/MapServer)

Population density at 30 arc seconds resolution (source LandScan http://www.ornl.gov/sci/landscan/index.shtml)

– Total surface area per country, including water bodies. http://wdi.worldbank.org/table/1.1

– Areas that are unsuitable for onchocerciasis as defined during the first REMO phase (see above)

– Geographic coordinates of all surveyed villages and for each surveyed village the percentage of examined adults who had palpable nodules, referred to as the “prevalence of nodules” or nodule prevalence.

– Surveyed area. This is the total area within 50 km from the nearest surveyed village. The threshold of 50 km corresponds to the maximum acceptable distance between sample villages as defined in the REMO manual [34]. Areas beyond 50 km from the nearest surveyed village are classified as non-surveyed. Excluded from both the surveyed and non-surveyed areas are unsuitable areas, national parks and water bodies.

### Geostatistical analysis

For probabilistic prediction of the true prevalence at both sampled and unsampled locations, a geostatistical model [44] was fitted in which conditional on the true prevalence P(x) at location x, the number of positives, Y, amongst a sample of N individuals follows a binomial distribution with N trials and “success” probability P(x). We used a standard logistic link function log(*P*(*x*)/(1 − *P*(*x*))) = *μ* + *S*(*x*), where *S*(*x*) is a low-rank approximation to a zero-mean isotropic Gaussian process [45]. For the main analysis, which excluded the spatially separate areas of Liberia and the island of Bioko, this process is defined as follows: (1) choose a discrete set of M points, say *X*_*j*_, over the region of interest; (2) represent *S*(*x*) as a weighted average of M independent, identically distributed zero-mean Gaussian variables *Z*_*j*_ with variance *σ*^2^, i.e. $\sum_{j=1}^{M}w\left(X_{j}-x\right)Z_{j}$, where the weights *w*(*X*_*j*_ − *x*) are chosen as functions of the great-circle distance, say *u*_*j*_, between *x* and each of the *X*_*j*_, so as to approximate the required correlation function of *S*(*x*). Note that, in this case, the variance *σ*^2^ does not represent variability on the logit scale since the range of variation of the *Z*_*j*_ variables is scaled by the kernel weights *w*(*X*_*j*_ − *x*). Following the procedure suggested by Rodriguez and Diggle [46], we used M = 10734 points *X*_*j*_ in a regular lattice at spacing 0.1 by 0.1 degrees and weights $w\left(X_{j}-x\right)=\text{exp}\left(-2\sqrt{2}u_{j}/\varphi \right)/\varphi$ to approximate a Matérn correlation function ([44] p.51-52) with scale parameter *φ* and smoothness parameter *κ* = 2.

In the separate analyses for Liberia and Bioko the dimensionality was much lower and there was no need of a low rank approximation of *S*(*x*). In these analyses, the zero-mean isotropic Gaussian process *S*(*x*) has Matérn correlation function, as previously defined, and variance *τ*^2^, which represents, unlike *σ*^2^, variation on the logit scale.

In each of the three analyses, model parameters were then estimated using the method of maximum likelihood based on the Laplace approximation method [47]. Maximum likelihood estimates, with associated 95% confidence intervals, of the geostatistical model parameters were for the main analysis (all REMO data excluding Liberia and Bioko) $\hat{\mu }=-2.451$ (−2.469, −2.432), ${\hat{\sigma }}^{2}=31.570$ (31.038, 32.112) and $\hat{\varphi }=65.208$ (64.993, 66.301) km. For Liberia the parameter estimates were $\hat{\mu }=-1.759$ (−1.779, −1.739), ${\hat{\tau }}^{2}=0.486$ (0.432, 0.547) and $\hat{\varphi }=57.945$ (52.151, 64.381) km. Finally for Bioko the estimates were $\hat{\mu }=-0.079$ (−0.283, 0.125), ${\hat{\tau }}^{2}=0.133$ (0.057, 0.310) and $\hat{\varphi }=1.950$ (0.535, 7.112) km. From the estimates of the scale parameters *ϕ* we determined that the range of the spatial correlation, defined as the distance at which the spatial correlation is 0.05 [44], is about 350 km for the main area, 311 km for Liberia and 10 km for Bioko. Hence pairs of observations within these distances in each of the three areas will show non-negligible spatial correlation.

The output from the fitted geostatistical model is a sample, of whatever desired size, from the joint predictive distribution of P(x), i.e. the conditional distribution of P(x) given all of the data, at locations x forming a regular grid at spacing 1 km over the entire surveyed area. A Monte Carlo Markov Chain method for conditional simulation of P(x) is used, based on the approach proposed by Giorgi *et al*. [48]. Any desired summaries of the predictive distributions can then be calculated and mapped. The two most relevant summaries for the current population are the mean of the predictive distribution of P(x) and the probability that P(x) exceeds 0.2 (20%), which corresponds to the operational criterion for delineating high-risk areas.

In order to deal with the high number of zero reported disease cases, we added zero prevalence data-points in areas free from the disease (ocean, deserts) when simulating from the predictive distribution of P(x). The fraction of added zeros corresponds to 5% of the total sample size beyond which very little impact was observed on the predicted prevalence surface. This approach decreases prevalence estimates in proximity of boundaries with areas free from the disease and avoids unrealistic high estimates of prevalence in such boundary areas. All computations were run on the High End Computer Cluster at Lancaster University, using the R statistical software environment [49].

### Estimation of population by endemicity level and number infected

The ‘at risk population’ of the surveyed areas in each APOC country was estimated by multiplying the surface of the surveyed area in the country with the country-specific average population density for CDTI projects. The latter was obtained for each APOC country by dividing the total population of the CDTI projects in the country in 2011 by the total surface area of these projects.

The nodule prevalence map was used to divide the surveyed area in each country into three endemicity classes with nodule prevalence of 0–4.5%, 5–19.9% and >20% respectively. The population in each class was estimated by multiplying the surface area with the average population density for CDTI projects in the country. For all surface calculations, the geographic coordinates were first projected using the ARCGIS (World) Cylindrical Equal Area projection.

In order to estimate the number of persons that would have been infected with *O. volvulus* by the year 2011 if there had been no onchocerciasis control, we used the recently published results of a study on the relationship between the prevalence of skin microfilaria in a village (all age groups combined) and the prevalence of palpable nodules in adult males in the same villages [42]. From this publication we used the main relationship for all study areas except one (Mbam), for which the pattern was different. This relationship was used to convert the 1 km resolution predicted nodule prevalence in adults, as generated during the geostatistical analysis, into the corresponding predicted prevalence of microfilaria for all ages combined. For each country, the predicted prevalence of microfilaria was then averaged over the total surveyed area and multiplied with the estimated at risk population of the surveyed areas in the country to obtain an estimate of the total number, T, infected with *O. volvulus* if there had been no onchocerciasis control. To obtain a confidence interval for this estimate, we sampled repeatedly from the joint predictive distribution of prevalence surface P(x), and from each sample calculated the corresponding estimate of T. Then, a 95% confidence interval for T is the range from the 2.5th to the 97.5th percentile of the empirical distribution of these estimates. For the APOC-wide total we used a similar procedure. Since nodule prevalence was modelled using three independent spatial processes with different means for the main area, Liberia and Bioko, we obtained a simulated sample for each from the joint predictive distribution of P(x), the estimated number of infected for the three areas separately and added these up. The 95% confidence intervals were then calculated from the resulting APOC-wide total distribution of T.

## Results

### Surveyed and excluded areas

The first step in the implementation of REMO was the exclusion of areas that were considered unsuitable for onchocerciasis transmission, and where, therefore, no nodule surveys were carried out. Also excluded at this stage were large water bodies and national parks that were considered uninhabited. The extent of the excluded areas in the different countries is summarised in Table 1. Large excluded areas covering more than 50% of the country surface were identified in Chad, Ethiopia, Kenya and Sudan. A description of the main unsuitable areas is provided in the companion paper [36].

**Table 1:** Extent of excluded and surveyed areas in the 20 APOC countries

**Country**	**Country surface (1000 km2)**	**Excluded area**		**Surveyed area**		**Non-surveyed area**	
		**1000 km2**	**%**	**1000 km2**	**%**	**1000 km2**	**%**
Angola	1,247	84	6.7	1,015	81.4	148	11.9
Burundi	28	4	13.0	24	87.0	0	0.0
Cameroon	475	25	5.2	430	90.5	21	4.3
CAR	623	129	20.6	448	71.9	46	7.4
Chad	1, 284	1, 027	80.0	257	20.0	0	0.0
Congo	342	59	17.1	271	79.3	12	3.5
DRC	2,345	183	7.8	2,053	87.6	109	4.6
Eq Guinea	28	4	15.2	23	80.4	0	0.0
Ethiopia	1,104	583	52.8	446	40.4	75	6.8
Gabon	268	19	7.3	191	71.5	57	21.2
Kenya	584	505	86.3	57	9.8	23	3.9
Liberia	96	1	0.8	96	99.2	0	0.0
Malawi	118	39	33.3	77	64.9	2	1.9
Mozambique	799	60	7.5	549	68.7	190	23.8
Nigeria	924	42	4.6	858	92.9	0	0.0
Rwanda	25	5	21.3	20	78.0	0	0.0
South Sudan	644	68	10.6	535	83.0	41	6.4
Sudan	1,861	1,516	81.4	346	18.6	0	0.0
Tanzania	947	380	40.1	393	41.5	174	18.4
Uganda	242	62	25.5	180	74.4	0	0.0
**Total**	**13,986**	**4, 793**	**34.3**	**8,270**	**59.1**	**898**	**6.4**

**Figure 1:**
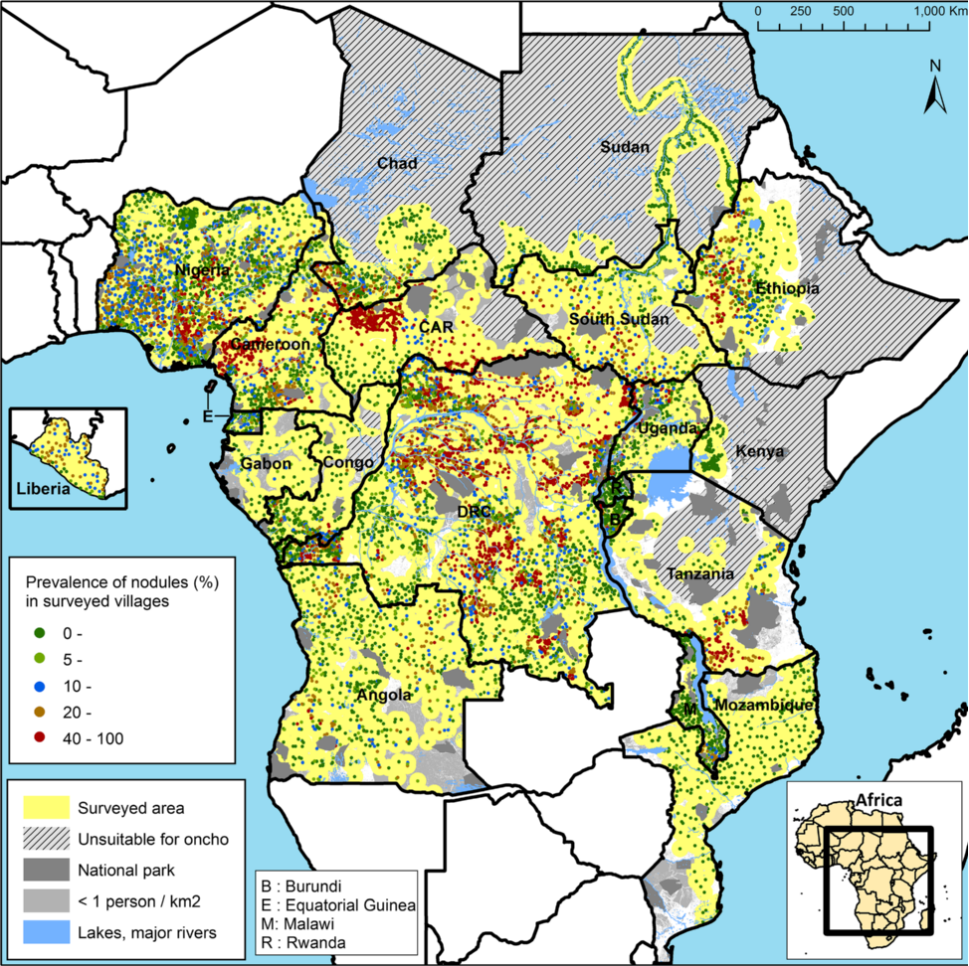
Map of the observed prevalence of palpable nodules in the 14,473 surveyed villages.

The remaining areas after the above exclusions were considered potentially endemic areas that needed to be surveyed for onchocerciasis. Table 1 shows for each country the extent of the areas that were surveyed and of the remaining non-surveyed area. In 8 countries all of the potentially endemic areas were surveyed. In 6 other countries, all (Central African Republic and Gabon) or nearly all (Angola, Cameroon, Congo and South Sudan) of the non-surveyed areas were uninhabited or had a very low population density of less than 1 person per km^2^. In only 2 of the remaining countries was the non-surveyed area more than 10% of the country surface: Mozambique (17%) and Tanzania (16%).REMO surveys were carried out in a total of 14,473 sample villages in the surveyed areas in the 20 APOC countries. Figure 1 provides a map showing the location and observed prevalence in the sample villages and the extent of the surveyed area.

### Map of the estimated prevalence of palpable nodules

**Figure 2:**
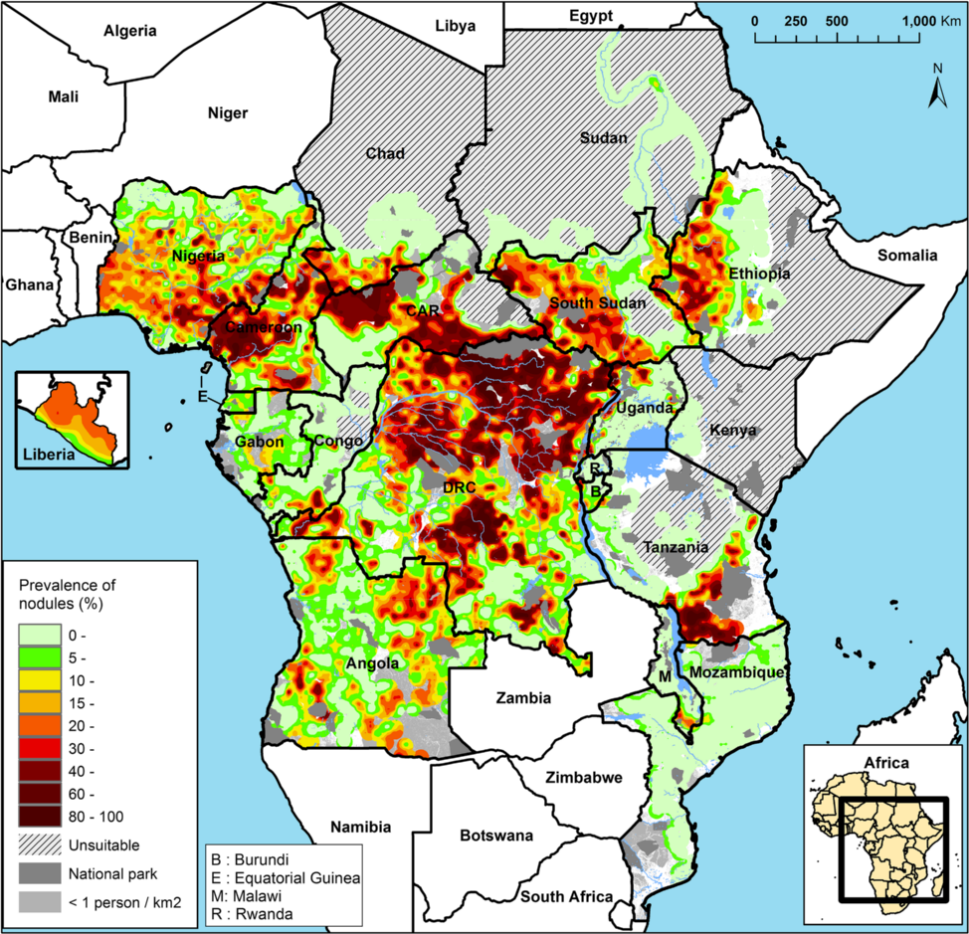
Map of the estimated prevalence of palpable nodules in the 20 APOC countries.

The model-based geostatistical analysis generated a map of the predicted prevalence of palpable nodules at 1 km resolution throughout the surveyed area in the 20 countries (see Figure 2). This map provides the best estimate of the geographic distribution of onchocerciasis endemicity levels based on the model based analysis of the REMO data. It shows substantial spatial variation in onchocerciasis endemicity levels. There are some vast areas where the endemicity levels are very high with the estimated prevalence of nodules exceeding 40%. A vast belt of hyperendemic onchocerciasis extends from the Democratic Republic of Congo through the west of South Sudan and the Central African Republic to Cameroon and south east Nigeria. There are also large hyperendemic foci in south Tanzania and west Ethiopia. On the other end of the endemicity scale there are several large areas where the prevalence of nodules is close to 0. This includes an area of some 500,000 km^2^ in North and Central Congo, South West of the Central African Republic and border areas of Cameroon, Gabon and the Democratic Republic of Congo where the results suggest that onchocerciasis is not endemic. Similar results were obtained for most of Mozambique, Malawi and Uganda, and large sections of Tanzania, Ethiopia, Sudan and Chad.

### Estimated population by endemicity level

Table 2 shows for each APOC country the classification of the surveyed areas into three endemicity classes with nodule prevalences of 0–4.9%, 5–19.9% and > 20% respectively. The table also gives the estimated population for these three categories for the year 2011. Overall, the predicted prevalence of nodules is greater than 20% over an area of 2.5 million km^2^ where an estimated 62 million people live. Another 77 million people are estimated to live in an area of 2.8 million km^2^ where the predicted nodule prevalence is between 5% and 20%. There are four countries, namely Cameroon, Central African Republic, Democratic Republic of Congo and Liberia, where more than 50% of the surveyed population live in areas where the predicted nodule prevalence is greater than 20%. In absolute numbers, the main countries are the Democratic Republic of Congo with 23.3 million people living in areas with more than 20% prevalence, Nigeria (14.3 million), Ethiopia (5.9 million), and Cameroon (5.2 million).

**Table 2:** Surveyed area and population by estimated nodule prevalence in the 20 APOC countries

**Country**	**Total surveyed area**			**Surface (1000 km2)**			**Estimated population (1000)**			**Estimated population as % of total population of surveyed area**		
	**Population per km2 in CDTi area**	**Surface (1000 km2)**	**Estimated Population (1000)**	**Prevalence of nodules 0-4.9%**	**Prevalence of nodules 5-19.9%**	**Prevalence of nodules ≥ 20%**	**Prevalence of nodules 0-4.9%**	**Prevalence of nodules 5-19.9%**	**Prevalence of nodules ≥ 20%**	**Prevalence of nodules 0-4.9%**	**Prevalence of nodules 5-19.9%**	**Prevalence of nodules ≥ 20%**
Angola	3.8	1,015	3,812	278	570	166	1,046	2,142	625	27.4	56.2	16.4
Burundi	341.0	24	8,252	11	11	2	3,821	3,760	671	46.3	45.6	8.1
Cameroon	24.2	430	10,389	64	149	217	1,555	3,599	5,234	15.0	34.6	50.4
CAR	5.2	448	2,312	135	81	232	697	418	1,198	30.1	18.1	51.8
Chad	21.6	257	5,557	152	51	54	3,286	1,099	1,171	59.1	19.8	21.1
Congo	34.8	271	9,432	194	62	15	6,750	2,159	523	71.6	22.9	5.5
DRC	22.1	2,053	45,391	318	683	1,052	7,040	15,089	23,262	15.5	33.2	51.2
Eq Guinea	22.0	23	433	3	18	1	54	298	81	12.4	68.8	18.7
Ethiopia	46.7	446	20,842	168	152	126	7,844	7,094	5,904	37.6	34.0	28.3
Gabon	NA	191	722	88	101	2	333	381	8	46.1	52.8	1.2
Kenya	NA	57	3,035	57	0	0	3,035	0	0	100.0	0.0	0.0
Liberia	30.2	96	2,884	0	42	53	10	1,269	1,604	0.3	44.0	55.6
Malawi	237.4	77	18,245	61	11	4	14,529	2,708	1,008	79.6	14.8	5.5
Mozambique	NA	549	9,889	483	65	1	8,694	1,170	25	87.9	11.8	0.2
Nigeria	65.3	858	56,016	175	463	220	11,440	30,239	14,336	20.4	54.0	25.6
Rwanda	NA	20	9,550	19	1	0	9,550	0	0	100.0	0.0	0.0
South Sudan	13.8	535	7,380	115	206	214	1,591	2,842	2,947	21.6	38.5	39.9
Sudan	14.6	346	5,053	331	13	2	4,841	190	23	95.8	3.8	0.4
Tanzania	19.4	393	7,631	221	66	106	4,289	1,276	2,065	56.2	16.7	27.1
Uganda	56.4	180	10,135	128	28	24	7,208	1,556	1,371	71.1	15.4	13.5
**Total**	**27.1**	**8,270**	**236,959**	**3,004**	**2,774**	**2,493**	**97,611**	**77,291**	**62,056**	**41.2**	**32.6**	**26.2**

### Priority areas for large scale treatment

**Figure 3:**
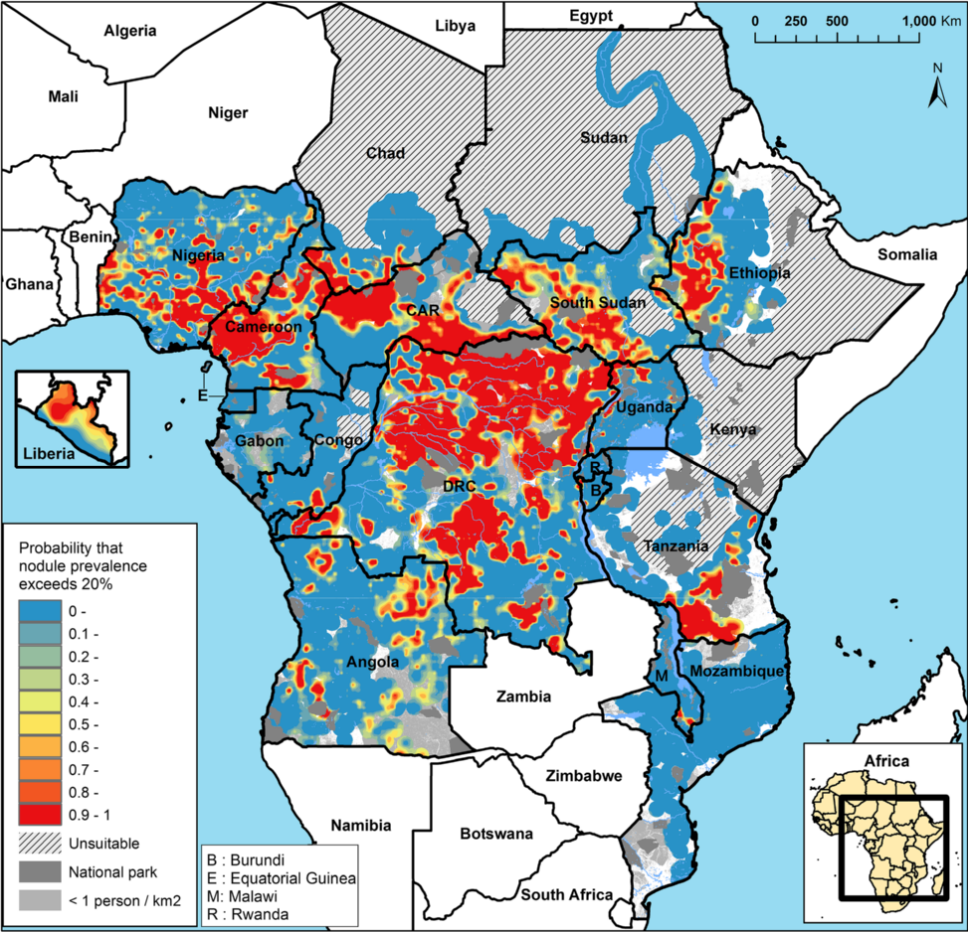
Map of the predictive probability that the local prevalence of nodules exceeds 20%.

The main objective of the REMO surveys was to identify priority areas for large-scale ivermectin treatment, i.e. areas where the prevalence of nodules is greater than 20%. The geostatistical analysis provides an objective method for defining such areas while taking the statistical uncertainty of the estimates into account. Figure 3 provides a map of the predicted probability that the local prevalence of palpable nodules exceeds the threshold of 20%. The map shows that for most of the surveyed area there is little uncertainty whether the prevalence of nodules exceeds the threshold or not: the probability is in most areas less than 0.1 (highly unlikely that the prevalence exceeds 20%) or greater than 0.9 (very likely that the prevalence is greater than 20%). Only for a few areas is the exceedance probability around 0.5, indicating that it is uncertain whether the prevalence exceeds the threshold. Most of these concern transition areas between high and low endemicity zones.

Table 3 provides a summary of the classification of the surveyed area according to the probability that the nodule prevalence exceeds 20%, and compares the results with those of the classification of high risk areas in the expert analysis described in the companion paper [36]. Using an exceedance probability of 0.5, it is estimated that the nodule prevalence exceeds 20% over a total surface of 2.3 million km^2^ with a population of 59 million people. However, these estimates are subject to considerable statistical uncertainty. Using an exceedance probability of 0.9 (highly likely that the nodule prevalence exceeds 20%), the corresponding population is only 36 million. Using an exceedance probability of 0.1, the population increases to as much as 90 million. The expert analysis reported in the companion article identified high risk areas with a total surface of 3.2 million km^2^ and a population of 84 million (this figure refers to high risk areas within the surveyed area; the experts also classified an additional 0.11 million km^2^ of unsurveyed area as ‘assumed’ high risk based on circumstantial evidence, giving a total of 3.3 million km^2^ of high risk areas and a population of 86 million reported in the companion paper). Table 3 shows the overlap between the two approaches. 98% of the priority areas for treatment that were identified with an exceedance probability of 0.9 in the geostatistical analysis were classified as high risk areas by the experts. The few differences between the two classification methods concerned minor differences in the delineation of boundaries of priority areas for treatment, with the expert analysis drawing boundaries according to river basins and the model based analysis, which currently does not include spatial information on rivers, drawing the boundaries often slightly wider. For the priority areas identified with the low exceedance probability of 0.1, the agreement with the experts was, unsurprisingly, poorer; only 80% of this area was classified as high risk by the experts.

**Table 3:** Comparison of priority areas for treatment identified by the two analytical approaches: expert analysis and geostatistical analysis

	**High risk (expert analysis)**								
**Probability that nodule prevalence exceeds 20% (geostatistical analysis)**	**Yes**			**No**			**Total**		
	**Surface 1000 km**^**2**^	**Population**		**Surface 1000 km**^**2**^	**Population**		**Surface 1000 km**^**2**^	**Population**	
		**(1000)**	**% of total**		**(1000)**	**% of total**		**(1000)**	**% of total**
> 0.9	1,440	35,447	98.0%	25	714	2.0%	1,465	36,161	100.0%
> 0.5	2,193	55,066	93.4%	152	3,877	6.6%	2,345	58,943	100.0%
> 0.1	2, 794	72, 479	80.4%	837	17, 682	19.6%	3,631	90,162	100.0%
**Total surveyed area**	**3,180**	**83,972**	**35.4%**	**5,089**	**152,986**	**64.6%**	**8,270**	**236,959**	**100.0%**

### Estimated number infected

The map of the predicted prevalence of nodules in adults in the 20 APOC countries, together with the recently published relationship between the prevalence of skin microfilaria and the prevalence of nodules, allowed the estimation of the total number of people that would have been infected with *O. volvulus* in the APOC countries if there had been no onchocerciasis control. The results of this analysis are shown in Table 4. It is estimated that overall 35.6 million people (95% confidence interval 35.1 to 36.1 million) would have been infected by 2011 if there had been no CDTI. Of those, 13.2 million are from the Democratic Republic of Congo and 8.5 million from Nigeria.

**Table 4:** **Estimated number of people that would have been infected with *Onchocerca volvulus *** **in the 20 APOC countries in 2011 if there had been no ivermectin treatment**

	**Population per km2 in CDTi areas**	**Surveyed area (1000 km**^**2**^**)**	**Rural population in surveyed area (1000)**	**Number infected with *O.volvulus (1000)***		
				**Estimate**	**Quantile 0.025**	**Quantile 0.975**
Angola	3.8	1,015	3,812	440	410	475
Burundi	341.0	24	8,252	658	603	717
Cameroon	24.2	430	10,389	2,810	2,674	2,956
CAR	5.2	448	2,312	592	562	624
Chad	21.6	257	5,557	551	516	591
Congo	34.8	271	9,432	512	442	605
DRC	22.1	2,053	45,391	13,155	12,869	13,462
Equatorial Guinea	22.0	23	433	58	55	62
Ethiopia	46.7	446	20,842	2,882	2,677	3,117
Gabon	NA	191	722	49	35	66
Kenya	NA	57	3,035	68	37	123
Liberia	30.2	96	2,884	554	515	596
Malawi	237.4	77	18,245	817	727	968
Mozambique	NA	549	9,889	330	275	398
Nigeria	65.3	858	56,016	8,510	8,292	8,750
Rwanda	NA	20	9,550	228	179	283
South Sudan	13.8	535	7,380	1,361	1,269	1,464
Sudan	14.6	346	5,053	58	50	68
Tanzania	19.4	393	7,631	1,061	975	1,152
Uganda	56.4	180	10,135	865	814	925
**Total**	**27.1**	**8,270**	**236,959**	**35,559**	**35,085**	**36,116**

As reported in the companion article, the prevalence of nodules was virtually zero in Kenya and Rwanda, suggesting that these two countries are non-endemic for onchocerciasis [36]. However, the necessarily imperfect calibration relationship between the prevalence of nodules and skin mf prevalence of Coffeng et al [42] shows that a zero nodule prevalence is compatible with skin mf prevalence between zero and about four percent. This explains why, in each of these two presumed non-endemic countries, our point estimate of the number of infected is approximately 2% of the population of the surveyed area.

## Discussion

The geostatistical analysis of the extensive REMO data for 14,473 surveyed villages has produced a detailed map of the pre-control geographic distribution of onchocerciasis endemicity levels in the 20 APOC countries. This map has been proven very valuable for onchocerciasis control and elimination.

Nearly all potentially endemic areas in the 20 APOC countries have been mapped for onchocerciasis. Of the total surface area of the 20 countries, 94% has been surveyed for onchocerciasis or classified as unsuitable for onchocerciasis transmission. By design, no surveys were done in the unsuitable areas. Although we have no reason to doubt the classification of unsuitability, we were not able to validate it with survey data. Most of the remaining 6% of unsurveyed area is either not populated or has a very low population density of less than 1 person per km^2^. It also includes a few zones for which it can reasonably be assumed that onchocerciasis is not endemic: the belts between surveyed and unsuitable areas in central Ethiopia and Kenya where the prevalence of nodules was zero in all neighbouring REMO villages; the unsurveyed areas in Mozambique south of latitude 18°S given that only 1 single nodule was detected in 37 villages surveyed below this latitude; and the coastal low lands of Tanzania where onchocerciasis vectors have never been reported [19,25,50]. Only for less than 1% of the total surface area of the 20 APOC countries may surveys still be needed to estimate the level of onchocerciasis endemicity. Hence the mapping of onchocerciasis in all potentially endemic areas in the APOC countries can be considered more than 99% complete.

The map of the pre-control prevalence of nodules that was generated in the geostatistical analysis predicts that before the start of CDTI, onchocerciasis was endemic in 18 of the 20 APOC countries. In Rwanda and Kenya (where onchocerciasis has been eliminated through vector control in the 1960s) the prevalence of nodules was virtually zero and these countries were classified as non-endemic. In Mozambique, the predicted prevalence of nodules was around zero throughout the country except for two small border areas with Tanzania and Malawi. In these two neighbouring countries there are hyperendemic onchocerciasis foci close to the border and this resulted in a predicted nodule prevalence of 15% to 20% just across the border in Mozambique. In the remaining 17 endemic countries, the endemicity levels of onchocerciasis varied significantly. There was a vast belt of hyperendemic onchocerciasis covering most of the Democratic Republic of Congo and extending across west Uganda, South Sudan, Central African Republic, Chad and Cameroon into Nigeria. In all of these countries the estimated nodule prevalence reached levels of over 40%, corresponding to skin microfilaria prevalence levels of about 60%. There were also large hyperendemic zones in Ethiopia and Tanzania with equally high prevalence levels. On the other hand, the estimated prevalence was close or equal to zero in most of Malawi, Uganda and Sudan, and in large sections of Burundi, Congo, Gabon, Tanzania, central Ethiopia and south-west Central African Republic. An intermediate pattern with low to medium prevalence levels was seen in the mainland of Equatorial Guinea and most of Angola. Overall, the predicted prevalence of nodules was greater than 20% over a surveyed area of 2.5 million km^2^ with an estimated population of 62 million, while the prevalence was between 5% and 20% over 2.8 million km^2^ with an estimated population of 77 million.

Beyond the APOC countries, onchocerciasis was known to be endemic in West Africa where the disease has been mapped by the OCP [2,51]). To the north of the surveyed area in the APOC countries are arid zones that are not suitable for Simulium vectors and which are therefore onchocerciasis free. For the same reason, Somalia is also considered onchocerciasis free even though the presence of *S. damnosum s.l.* (though not the disease) was reported from one area in the 1950s [25]. To the south of APOC, all countries except one are located below the most southern latitude at which onchocerciasis has ever been reported. The exception is Zambia. Since Zambia is not a participating country of APOC, REMO surveys have not been done in this country. In the literature there is only one report from 1983 of an infection with *O. volvulus* in a child [52], otherwise onchocerciasis has never been reported from Zambia. However, in the absence of systematic survey data, we cannot be certain that the country is onchocerciasis free, especially for some border areas.

Compared to the historical information on the geographic distribution of onchocerciasis in the APOC countries, the nodule prevalence map is a significant advance. The WHO Expert Committee on Onchocerciasis Control of 1995 produced a provisional map of endemic onchocerciasis in Africa on the basis of information available at that time [1]. Much of the area that the Committee identified as endemic for onchocerciasis has been confirmed endemic in the geostatistical analysis of the REMO data. However, there were several large areas that the Committee labeled as non-endemic but that were shown to have medium to high prevalence levels in the nodule prevalence map. These include endemic foci in North Nigeria, South Cameroon, South Sudan, much of Angola, and several large hyperendemic zones in the Democratic Republic of Congo where the prevalence of nodules exceeded 50%-80%. Conversely, several areas labeled as endemic by the Committee had an estimated nodule prevalence around zero, e.g. the zone in the south-west of the Central African Republic and the north of Congo.

A second limitation of the historical data was the lack of information on onchocerciasis endemicity levels for most areas. The REMO surveys filled this gap and generated detailed information on onchocerciasis endemicity that was critically important for APOC to identify priority areas for ivermectin treatment, i.e. areas where the prevalence of nodules exceeded 20%. Wherever REMO data became available, they were subjected to an expert analysis that delineated high risk areas where the prevalence of nodules was greater than 20% and where CDTI was subsequently implemented to control the disease as a public health problem. The results of the expert analysis are described in the companion paper. The expert analysis used a standard methodology to analyse the REMO data within the context of other relevant geographic information. The ability to take data from multiple sources into account was a strength of this methodology but a perceived weakness was its subjective component: the experts’ interpretation of the information. The geostatistical analysis involves an objective statistical method that can take statistical uncertainty into account in the decision making process on priority areas. Given these fundamental differences between the two analytical approaches, it was of interest to compare their results.

Using the geostatistical analysis it was predicted that the local prevalence of nodules was equal to or greater than the threshold of 20% over a total surface area of 2.5 million km^2^ with a population of 62 million people. This is less than the high risk area of 3.2 million km^2^ with a population of 84 million identified in the expert analysis. However, in contrast to the expert analysis, the geostatistical estimate has the advantage that it is accompanied by an estimate of its statistical uncertainty. Taking into account the probability that the local prevalence exceeds the 20% threshold, the surface area ranges from 1.5 million km^2^ to 3.6 million km^2^ for exceedance probabilities of 0.9 and 0.1 respectively. For exceedance probabilities of 0.9, nearly all the surface area classified as having a prevalence of nodules > 20% was also classified as high risk in the expert analysis. For the low exceedance probability of 0.1, there was agreement with the expert analysis for only 80% of the area classified as exceeding the 20% threshold. The results indicate that the two methods gave comparable results for areas where the prevalence of nodules clearly exceeds (i.e. exceedance probability > 0.9) the threshold of 20%, and where ivermectin treatment is therefore needed to control onchocerciasis as a public health problem, but that there is some disagreement for borderline areas where the prevalence of nodules fluctuates around or below 20%. We conclude that the expert analysis has correctly identified all areas for which there is strong evidence that ivermectin treatment is needed to control onchocerciasis as a public health problem. It also includes many borderline areas for which the evidence of high risk is less strong, but this has been considered justified for ethical reasons so as not to exclude isolated high-risk communities from treatment [36].

The geostatistical analysis has also been used to estimate the total number of people that would have been infected with *O. volvulus* in the 20 APOC countries if there had been no CDTI. Based on the nodule prevalence map and the recently published quantification of the relationship between the prevalence of skin microfilaria and the prevalence of onchocercal nodules [42], we estimate that some 35.6 million people (95% confidence interval 35.1 to 36.1 million) would have been infected by the year 2011 if there had been no CDTI. This estimate is significantly higher than the most commonly quoted estimate from the WHO Expert Committee on Onchocerciasis Control which estimated that in 1995 a total of 17.7 million people were infected globally, of which 15.0 million lived in APOC countries [1]. Using an annual rural population growth rate of 2.2% for the APOC countries [53], our estimate of 36 million infected for 2011 corresponds to 25 million infected in 1995, i.e. 10 million more than the previous WHO estimate for the APOC countries. This difference is not surprising given that REMO identified many new endemic areas and generated prevalence estimates for all areas. However, compared to other, more recent estimates our figure appears low. Coffeng *et al.*[54] reported an estimate of 32 million people infected in the APOC countries in 1995, and Remme *et al.*[55] estimated 37 million people infected globally in 1995 and also about 32 million for the APOC countries. These estimates are also largely based on APOC’s REMO data. The difference with our estimate is mainly due to two methodological factors. One concerns the formula used to quantify the relationship between the prevalence of microfilaria and the prevalence of nodules. We used a formula from a recently published analysis of data from West, Central and East Africa [42] which predicts a lower prevalence of microfilariae for a given prevalence of nodules than the formulas used previously. The second factor concerns the way the REMO sampling design has been taken into account. The previous estimates assume that sample villages were selected randomly from a given area. However, in the REMO sampling method villages are selected spatially at regular distances along rivers with potential breeding sites and at lower sampling density between rivers. Because of this design, the selection of villages to be surveyed is biased towards villages with a high endemicity level close to breeding sites and this bias may have resulted in an overestimate of the number infected in previous studies. The current geostatistical analysis partially corrects for this bias by taking into account the spatial distribution of the survey data. Specifically, in estimating the total number infected, one effect of the spatial correlation is that the observed prevalence from an isolated surveyed village acts as a proxy for the results that would have been obtained had surrounding villages also been surveyed, and therefore has greater influence than any one of a number of surveyed villages at mutually close locations. This results in a discrepancy between the crude average prevalence and the spatially averaged modelled prevalence.

A possible improvement of the geostatistical analysis of the REMO data would be to include relevant geographical covariates in the geostatistical model [56], such as the distance to the nearest river with breeding sites, local *Simulium* species and vegetation. This will not be easy as the distribution of the different Simulium species is not well known for most areas while the identification of rivers with potential for *Simulium* breeding is a challenge, especially in forest areas. However, recent progress in the development of a remote sensing model to identify *S. damnosum s.l.* breeding sites in Africa appears promising [57]. If this approach can be made to work also in forest areas, and if the cost of its large scale application can be reduced, it should be possible to improve the nodule prevalence map by including in the model the distance to the nearest potential *S. damnosum* breeding site as identified by remote sensing data. Another possible improvement of the model concerns predictions in areas where the prevalence is zero. A common feature of prevalence survey data, here and elsewhere, is an excess of zeros by comparison with the best-fitting binomial distribution. In a spatial setting, this zero-inflation can be artificial; for example, it could be the result of over-sampling in low-prevalence areas. In principle, geographical covariate information could again be used to model genuine zero-inflation [58]. In our analysis, we dealt with this by adding dummy zero prevalence data at points within areas known to be disease-free (eg deserts and large water-bodies), thereby ensuring that our estimated prevalence approaches zero at the boundaries of each of these areas. We intend to develop an extended model which treats zero-inflation as a second spatial stochastic process for applications where areas of true zero prevalence are not known beforehand and prediction of such areas is important. One such application is the use of the REMO data for helping to revise ivermectin treatment boundaries for the purpose of onchocerciasis elimination. Finally, bias would arise if implementers deliberately sampled communities whose prevalence was atypical of their general localities, a phenomenon called preferential sampling. Correcting for the effects of preferential sampling is difficult unless it can be explained by measured covariates such as distance to the nearest river in the case of onchocerciasis [56].

The original objective of REMO was to identify target areas for ivermectin treatment with the aim of controlling onchocerciasis as a public health problem. In recent years evidence has emerged that in the long term onchocerciasis infection and transmission can even be eliminated with CDTI [59-61]. Based on this new evidence, APOC has adopted an additional objective to eliminate onchocerciasis where feasible [62]. Because of this paradigm shift, the target areas for CDTI are currently being revised to include all areas with local onchocerciasis transmission. The nodule prevalence map provides the starting point for determining the new treatment boundaries. Furthermore, the number of years of ivermectin treatment that is required to achieve elimination depends strongly on the local endemicity level [63]. Information on pre-control endemicity levels is therefore essential for the correct interpretation of the results of epidemiological evaluations of the impact of CDTI on onchocerciasis infection levels, and for the prediction of the remaining number of years of CDTI needed in a given area [40]. This information is now also available for all CDTI areas from the nodule prevalence map.

## Conclusions

APOC is close to achieving the objective of controlling onchocerciasis as a public health problem throughout the APOC countries, and the REMO data and nodule prevalence maps have played an essential role in targeting treatment where needed to achieve this objective [64]. Following the shift from onchocerciasis control to onchocerciasis elimination, the nodule prevalence map will continue to play an important role and help with adjusting treatment boundaries, interpreting epidemiological evaluation data on progress towards elimination and predicting when elimination will be achieved in different areas. REMO was a major undertaking but it has been worthwhile and the results have been very valuable for onchocerciasis control and elimination in Africa.

## Competing interests

The authors declare that they have no competing interests.

## Authors’ contributions

MN, AT, HZ, UVA and JHFR were involved in the design and implementation of REMO. HZ was responsible for data processing. PJD, HZ, EG and JHFR conceptualized the geostatistical analysis. PJD, EG, HZ, AT and JHFR did the analysis. JHFR drafted the manuscript and all authors contributed to and approved the final manuscript.
